# A retrospective analysis of the clinicopathological features and prognostic value of MAPK12 protein expression in diffuse large B-cell lymphoma

**DOI:** 10.1007/s12094-024-03515-3

**Published:** 2024-05-21

**Authors:** Yue Liu, Han Zhang, Shu Zhao, Yue Zhang

**Affiliations:** Department of Medical Oncology, Harbin Medical University Cancer Hospital, Harbin, China

**Keywords:** MAPK12, Diffuse large B-cell lymphoma, Immunohistochemistry, Prognosis

## Abstract

**Purpose:**

Mitogen-activated protein kinase 12 (MAPK12), also known as p38γ, is a member of the p38 MAPK family and plays a crucial role in tumor occurrence and invasion. However, there is still uncertainty regarding MAPK12 involvement in diffuse large B-cell lymphoma (DLBCL).

**Methods:**

Our study investigated the expression of MAPK12 mRNA in various types of cancer using bioinformatic analysis. Furthermore, we performed immunohistochemistry (IHC) to detect the expression of MAPK12 in patients with DLBCL and compared clinical indicators and survival rates.

**Results:**

We found that the high expression rate of MAPK12 was 43.1% in DLBCL patients. Several clinical indicators, including IPI scores, Hans classifications, LDH levels, and Ki-67 expression were closely associated with MAPK12 expression. Survival analysis revealed that higher expression of MAPK12 was significantly correlated with shorter progression-free survival (PFS) and overall survival (OS) in DLBCL patients. In addition, both univariate and multivariate analyses revealed IPI score, MAPK12 expression, and rituximab use as the independent OS risk factors (*P* < 0.05). To explore the functional role of MAPK12 in DLBCL, weighted gene co-expression network analysis (WGCNA) and gene ontology (GO) were used to confirm the involvement of MAPK12 in the regulation of type II interferon production, positive regulation of lymphocyte proliferation, and other related biological processes.

**Conclusion:**

DLBCL patients have poor prognoses when MAPK12 levels are high, which is expected to be a therapeutic target and prognostic factor.

## Introduction

The most prevalent subtype of non-Hodgkin lymphoma (NHL) is diffuse large B-cell lymphoma (DLBCL), and it originates from lymph nodes, extranodal organs, or tissues and can also transform from indolent lymphomas[1, 2]. There has been a significant advancement in DLBCL research with the identification of biologically distinct subtypes. In most cases, DLBCL is classified as GCB-like (germinal center B cell), ABC-like (activated B cell), and unclassified (UC)[3, 4]. A significant increase in cure rates for DLBCL and a significant improvement in PFS and OS have been achieved with the use of R-CHOP. According to Hans classification, the GCB subtype has a better prognosis than the non-GCB subtype, especially after R-CHOP treatment [5]. Although rituximab considerably improves prognosis, approximately 40% of patients still experience recurrent or resistant disease [6]. Hence, the exploration of novel disease targets and the refinement of existing prognostic index systems represent pressing clinical imperatives.

MAPK12, or p38γ, is a serine/threonine protease consisting of 367 amino acids. It belongs to the p38 family, which comprises three additional subtypes: p38α, p38β, and p38δ. There exists a high degree of similarity among the amino acid sequences of each subtype [7]. Apart from their pivotal involvement in numerous biological processes, they also have a significant impact on the genesis and progression of malignancies [9]. For example, p38α and p38δ is highly expressed in breast cancer, head and neck squamous cell carcinoma, skin cancer tissues, and other tumors [10]. In endometrial cancer, p38β promotes tumor cell growth by preventing apoptosis[11]. MAPK12 (or p38γ) plays a crucial role in various physiological and pathological processes, such as proliferation, metabolism, apoptosis, inflammation, regulating cell cycle, and innate immune response [12–14]. In previous studies, MAPK12 has been implicated in the development and progression of various tumor types. For instance, in breast cancer, Xu et al. discovered that overexpression of MAPK12 promotes epithelial-mesenchymal transition (EMT) [15]. Specifically, in triple-negative breast cancer, overexpression of MAPK12 facilitates the expansion of tumor stem cell and cell transformation, thereby accelerating cancer progression[15]. The overexpression of MAPK12 in osteosarcoma tissues and cells enhances cell growth, proliferation, and migration and promotes the progression of osteosarcoma [16]. As a result of targeting MAPK12 in colorectal cancer can reduce tumor proliferation and induce apoptosis [17]. The molecular mechanisms and carcinogenic effects of MAPK12 in DLBCL remain elusive, despite numerous studies demonstrating its involvement in tumorigenesis. Hence, it is crucial to investigate MAPK12 expression in DLBCL patients and understand its role in the disease.

The leading goal of the current study was to examine the expression of MAPK12 using immunohistochemical staining in a relatively large population of DLBCL patients. The results were compared to clinicopathological characteristics and survival rates. Our exploration of MAPK12 as a potential treatment target for DLBCL further underscores its potential involvement in various biological processes within the disease, as revealed by bioinformatics methods.

## Methods

### Processing of data and analysis of differential expressions

The Cancer Genome Atlas database (TCGA; https://portal.gdc.cancer.gov/) retrieved 11,057 samples for RNA sequencing of 33 different cancer types. These data were standardized, and a differential expression analysis for MAPK12 was completed using the R/ggpubr package to draw a box plot. The expression of MAPK12 in TCGA cancers was further examined using GEPIA (http://gepia.cancer-pku.cn/index.html), with the matching TCGA normal and GTEx data used as controls. Additionally, four cohorts of DLBCL patients—GSE56315, GSE32018, GSE11318, and GSE10846—were compiled from the Gene Expression Omnibus (GEO) database, along with the Series Matrix File and GPL6480, GPL570, to analyze variations in MAPK12 expression across different tissue types and investigate the association between MAPK12 mRNA levels and prognosis.

### Patients

From November 2008 to April 2018, 153 patients diagnosed with DLBCL at the Harbin Medical University Cancer Hospital participated in this study. All cases were evaluated according to the World Health Organization criteria (WHO 2017) and received a standardized treatment regimen of CHOP/CHOPE or R-CHOP/CHOPE for a minimum of four cycles. Each patient included in the study had comprehensive clinicopathological and follow-up data, encompassing age, gender, clinical stage, International Prognostic Index (IPI) score, Hans classification, and other relevant factors. Table 1 displays the primary patient characteristics. Postoperative survival was calculated from the day of diagnosis, and follow-up was conducted until death or January 2022. The primary endpoints of this study were overall survival (OS) and progression-free survival (PFS). The ethics committee of Harbin Medical University Cancer Hospital approved this study, and the principles of the Helsinki Declaration were followed. The consent of all patients was obtained.

**Table 1:** Relationship between clinicopathological factors and MAPK12 expression levels in 153 DLBCL patients

Characteristics	Cases N (%)	MAPK12 expression		P value
		Low	High	
Age (years)				0.070
≤ 60	109(71.2%)	67(77.0%)	42(63.6%)	
> 60	44(28.8%)	20(23.0%)	24(36.4%)	
Gender				0.254
Male	73(47.7%)	45(51.7%)	28(42.4%)	
Female	80(52.3%)	42(48.3%)	38(57.6%)	
Extranodal invasion				0.417
< 2	125(81.7%)	73(83.9%)	52(78.8%)	
≥ 2	28(18.3%)	14(16.1%)	14(21.2%)	
Tumor size				0.263
< 10 cm	137(89.5%)	80(92.0%)	57(86.4%)	
≥ 10 cm	16(10.5%)	7(8.0%)	9(13.6%)	
Hans classification				0.024^*^
Non-GCB	79(51.6%)	38(43.7%)	41(62.1%)	
GCB	74(48.4%)	49(56.3%)	25(37.9%)	
ECOG score				0.794
< 2 score	119(77.8%)	67(77.0%)	52(78.8%)	
≥ 2 score	34(22.2%)	20(23.0%)	14(21.2)	
B symptoms				0.926
Absent	120(78.4%)	68(78.2%)	52(78.8%)	
Present	33(21.6%)	19(21.8%)	14(21.2%)	
Ann Arbor stage				0.775
I + II	97(63.4%)	56(64.4%)	41(62.1%)	
III + IV	56(36.6%)	31(35.6%)	25(37.9%)	
IPI score				0.040^*^
0–2	104(68.0%)	65(74.7%)	39(59.1%)	
3–5	49(32.0%)	22(25.3%)	27(40.9%)	
LDH				0.041^*^
≤ 246	84(54.9%)	54(62.1%)	30(45.5%)	
> 246	69(45.1%)	33(37.9%)	36(54.5%)	
Ki-67				0.035^*^
≤ 70%	61(39.9%)	41(47.1%)	20(30.3%)	
> 70%	92(60.1%)	46(52.9%)	46(69.7%)	
Therapy				
CHOP	77(50.3%)	44(50.6%)	33(50%)	0.944
R-CHOP	76(49.7%)	43(49.4%)	33(50%)	

^*^p ≤ 0.05

### Immunohistochemistry

A 3-μm-thick formalin-fixed paraffin-embedded segment from each biopsy specimen obtained from 153 patients with DLBCLs was prepared for immunohistochemistry analysis. In brief, the tissue was sliced into representative pieces and placed on slides with poly-L-lysine coating. Prior to immunohistochemical staining, the specimens underwent xylene dewaxing, hydration in anhydrous ethanol, and antigen retrieval using microwave. The slides were sealed with a blocking solution, and any extra liquid was discarded after washing in phosphate-buffered saline (PBS) to reduce specific binding. An overnight incubation at 4℃ with a primary antibody (20184–1-AP, dilution 1:300; Proteintech, China) was performed. As a negative control, PBS was used to replace the primary antibody to maintain specificity. Subsequently, the slides were treated with a secondary antibody (ZSGB-BIO, China) that was HRP-labeled for an additional 30 min at room temperature. The slides were then dehydrated, cleaned, and mounted after being counterstained with hematoxylin.

### Determination of MAPK12

The MAPK12 IHC score was independently assessed by two experienced pathologists. The IHC score was calculated using a semi-quantitative approach that considered both the staining intensity and the proportion of staining positive cells. Based on the hue of the immunological response, the degree of staining was divided into four categories: negative (0), light brown (1), brown (2), and tan (3). The number of cells that stained positively was determined and given the following scores: 0 for 0–5%,1 for 6–25%, 2 for 26–50%, 3 for 51–75%, and 4 for 76–100%. The total score (maximum 0–12) was calculated by multiplying the two scores. For example, scores 0–2 were considered negative expressions (-, noted as 0); 3–5 were considered mild positive expressions (+ , indicated as 1); 6–8 were considered moderate positive expressions (+ + , indicated as 2); 9–12 were deemed strong positive expressions (+ +  + , indicated as 3). Low expression was characterized by scores as 0 and 1, while scores indicated high expression as 2 and 3.

### Weighted gene co-expression network analysis (WGCNA) and gene ontology(GO) of MAPK12 expression

From The Cancer Genome Atlas database (TCGA; https://portal.gdc.cancer.gov/), we downloaded RNA-seq data for DLBCL. We collected 48 samples of DLBCL tissue. The "WGCNA" program was used to create genetic co-expression modules from DLBCL gene expression data. The topological overlap matrix (TOM) and the accompanying dissimilarity degree (1-TOM) were computed using a soft power value of 9. The adjacency matrix for the 1-TOM matrix was then clustered to group the genes. Using a dynamic tree-cut method, we identified the gene modules in the dendrogram with a minimum of 50 genes in each module. Module-trait heat maps were used to calculate the correlation between module-trait and MAPK12 expression. The DLBCL database, GSE10846, and GSE11318 were retrieved from the GEO database at https://www.ncbi.nlm.nih.gov/geo/. Differentially expressed genes (DEGs) were filtered using the R/limma package, with a threshold of |log2FoldChange|≥ 1.5 and *P* ≤ 0.05. The volcano plot for the DEGs was then created using the R/ggplot 2 package.

Following the combination of the GSE10846 and GSE11318 datasets with the TCGA bright blue module, 21 genes were identified. To explore their association with MAPK12, heat mapping was conducted using an online tool ( http://www.heatmapper.ca/expression/). Additionally, the R/enrichplot package was utilized to conduct the GO enrichment study.

### Statistical analysis

We evaluated the association between MAPK12 expression and the clinicopathologic characteristics of the DLBCL patients using the χ2 test. Survival analyses were conducted in GraphPad Prism 9 using the log-rank (Mantel–Cox) test. To analyze MAPK12 differential expression and create scatter plots, we employed the *t* test. The expression levels of MAPK12 were used to divide the patients into low and high groups. The Cox regression approach was used to obtain hazard ratios (HRs) with 95% confidence intervals (CIs) in univariate and multivariate studies. The groups were compared using the Student's *t* test. All statistical analyses were performed using SPSS 27.0 (IBM Corp., Armonk, NY, USA) software. Statistical significance was defined as a two-tailed *P*-value < 0.05.

## Results

### Differential expression of MAPK12 in pan-cancer and DLBCL

**Fig. 1:**
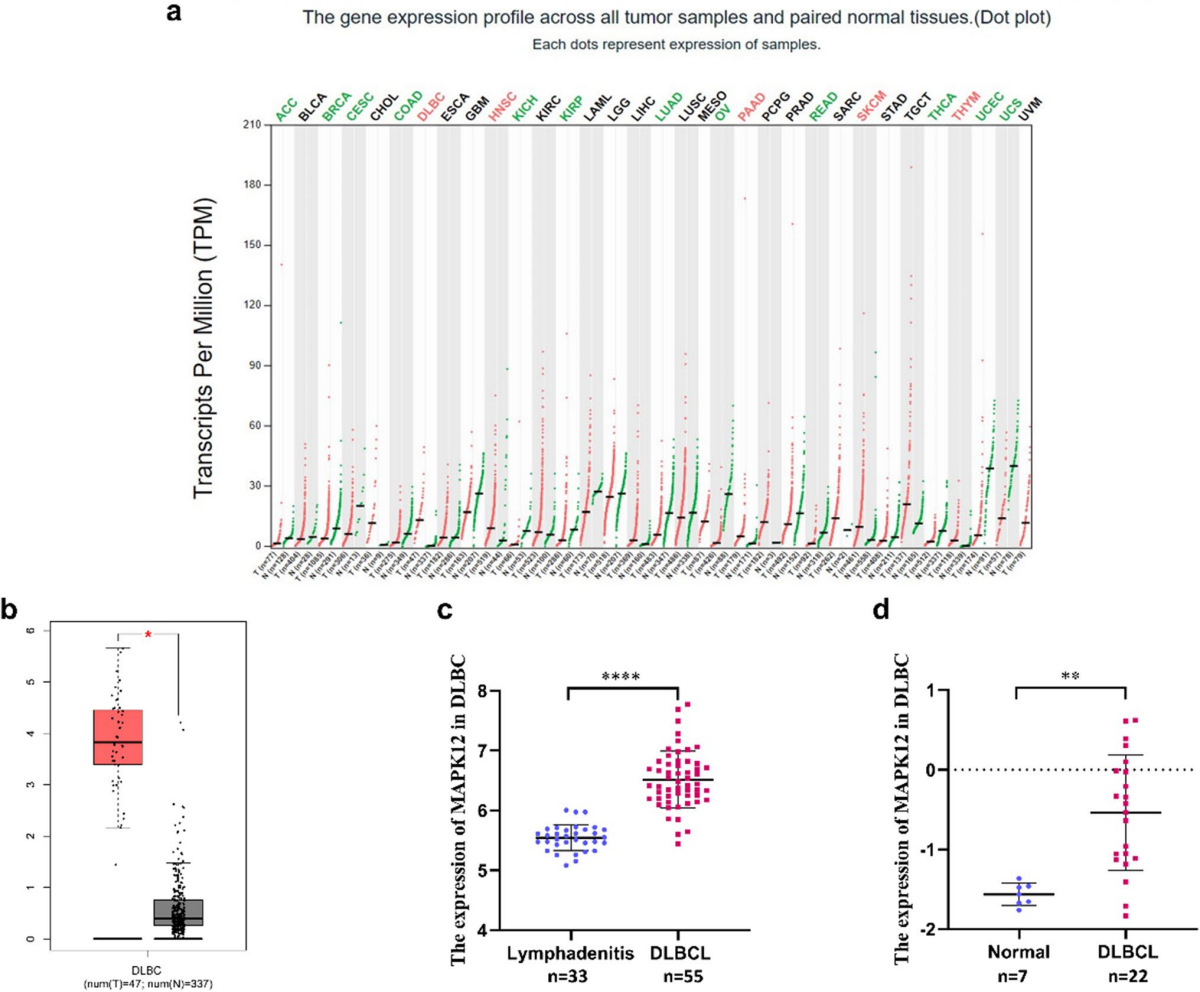
Differential expression of MAPK12 in pan-cancer and DLBCL. **a.** GEPIA database showing the expression of MAPK12 in tumor and normal tissues. **b.** In database GEPIA, MAPK12 expression was elevated in DLBCL. **c.** In GSE56315, the expression of MAPK12 in DLBCL and lymphadenitis was significantly different. **d.** In GSE32018, the expression of MAPK12 in DLBCL and lymph nodes was significantly different. (*p ≤ 0.05,**p ≤ 0.01,***p ≤ 0.001, ****p ≤ 0.0001)

MAPK12 is highly expressed in various tumor tissues as depicted in Fig. 1a. We found that MAPK12 expression in DLBCL was significantly higher compared to lymph nodes. This finding was further validated by data from the GEO database (Fig. 1b-d).

## Patient characteristics

A total of 153 DLBCL samples were obtained from Harbin Medical University Cancer Hospital. We summarized the baseline characteristics in Table 1 to demonstrate the relationship between MAPK12 expression and clinicopathologic characteristics. Among the patients, 109 (71.2%) were aged ≤ 60 years and 44 (28.8%) were aged > 60 years. Of the 153 patients, 73 (47.7%) were males. Elevated lactate dehydrogenase (LDH) levels were observed in 69 patients (45.1%), while 33 patients (21.6%) exhibited B symptoms. 97 patients (63.4%) had limited-stage disease (Ann Arbor stage I/II), and 56 patients (36.6%) had advanced-stage disease (Ann Arbor stage III/IV). An intermediate IPI of high-risk factors was seen in 32.0% of patients. In addition to standard chemotherapy, 76 patients (49.7%) received rituximab therapy.

## MAPK12 expression in DLBCL and its relationship to clinical parameters

**Fig. 2:**
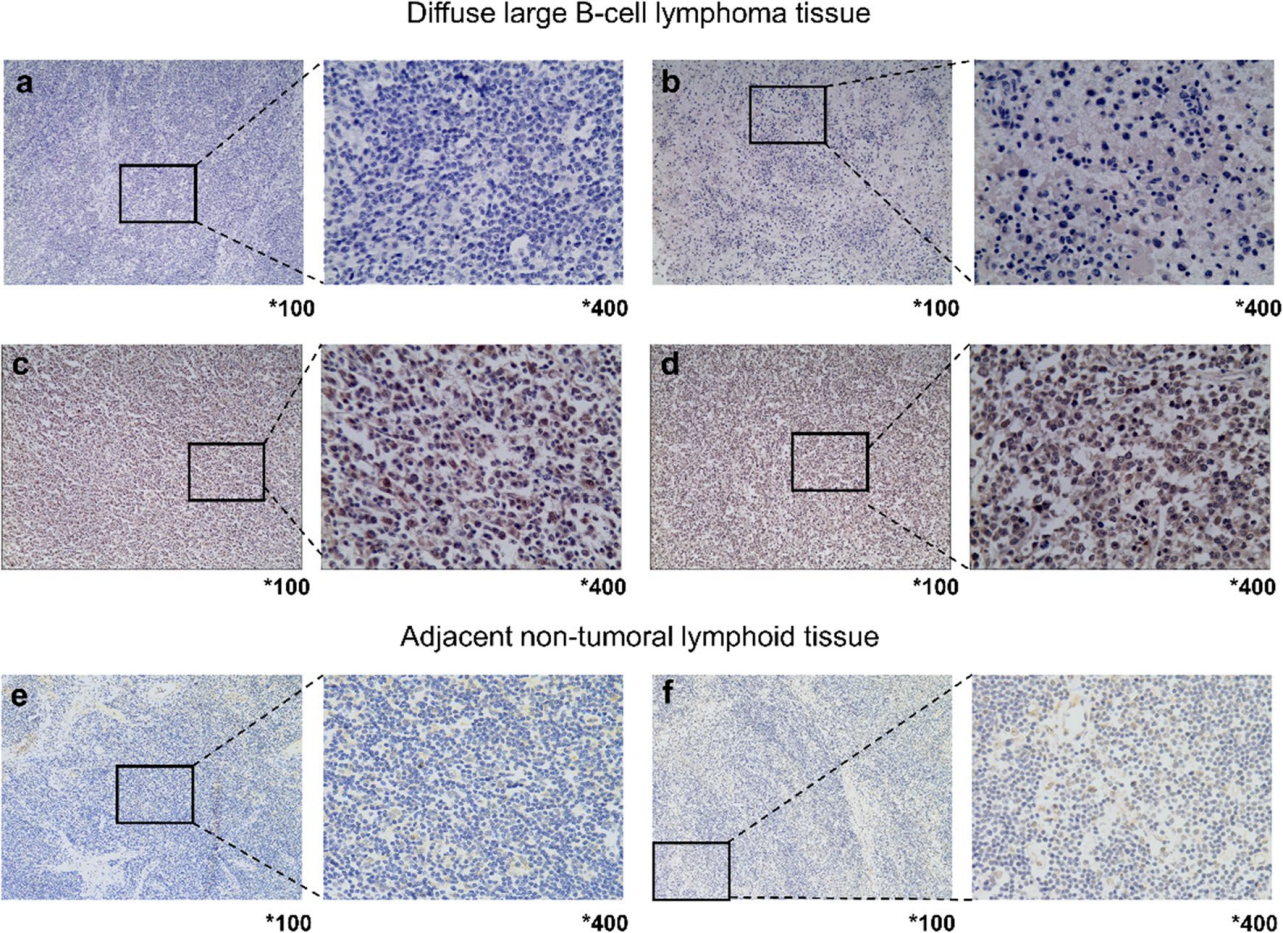
Typical IHC pictures of the expression of MAPK12 in DLBCL and adjacent non-tumoral lymphoid tissue. **a.** Negative, **b.** slightly positive, **c.** moderately positive, and **d.** highly positive MAPK12 expression in DLBCL tissues. **e.** Negative and **f.** slightly positive MAPK12 expression in adjacent non-tumoral lymphoid tissue

The expression of MAPK12 in DLBCL was investigated by IHC using tissue samples obtained from 153 patients. The positive staining of MAPK12 was predominantly observed in the cytoplasm (Fig. 2). MAPK12 staining intensities varied from colorless to pale brown, brown, and tan, in a diffuse and scattered pattern. Out of the 153 DLBCL specimens, 87 patient samples (or 56.9%) were categorized as having low MAPK12 expression (Fig. 2a-b), while the remaining 66 samples (or 43.1%) were categorized as having high MAPK12 expression (Fig. 2c-d). MAPK12 was low expression in 72 adjacent non-tumoral lymphoid tissues (or 100%) (Fig. 2e-f). There was a strong correlation between MAPK12 expression and higher IPI scores, non-GCB subtype, lower LDH levels, and higher Ki-67 expression in the data previously accessible. Nevertheless, no statistically significant correlations were observed between MAPK12 expression and gender, age, number of extranodal invasions, tumor size, ECOG score, B symptoms, Ann Arbor stage, or therapy.

## Relationship between MAPK12 expression and survival in DLBCL

**Fig. 3:**
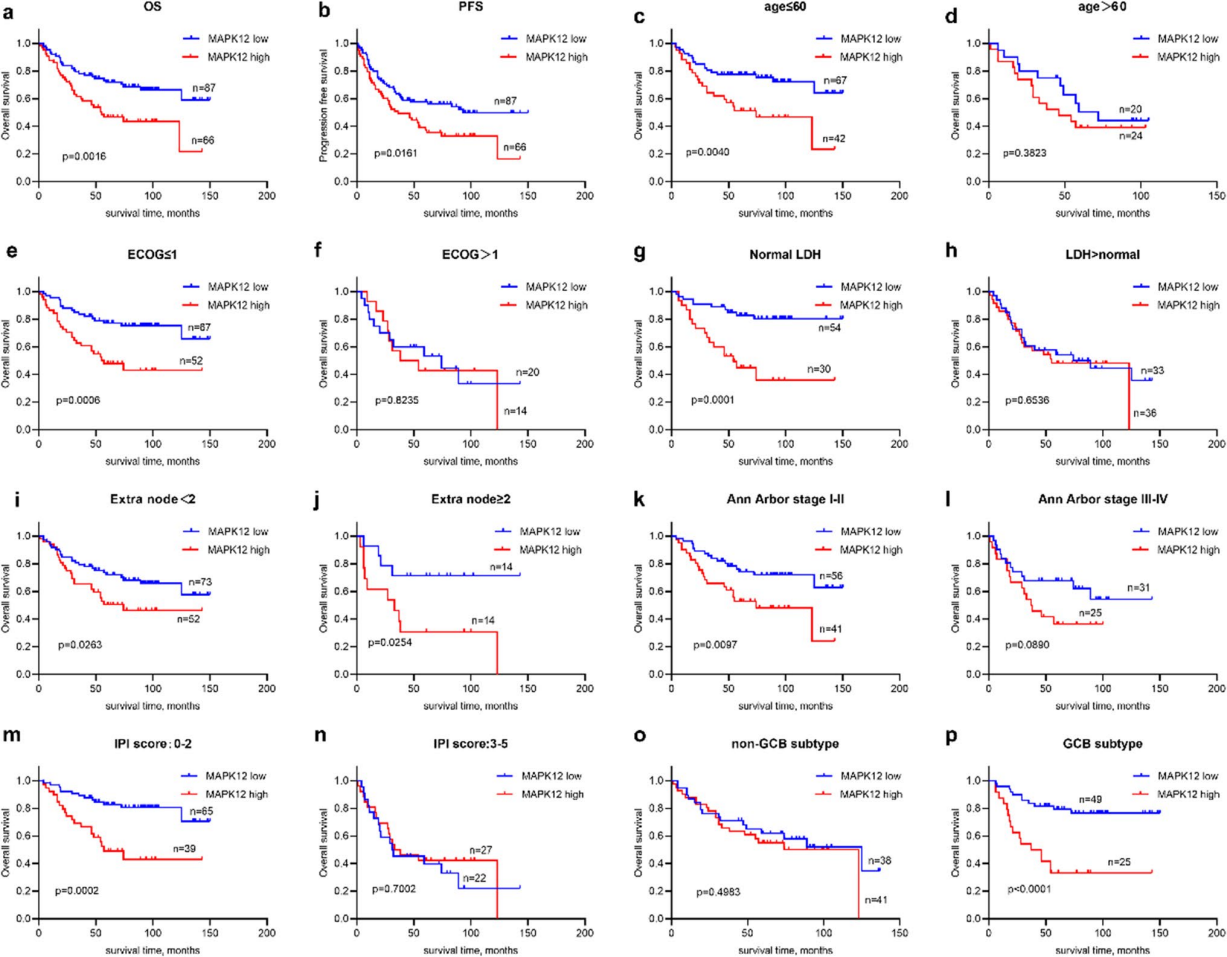
A correlation between low survival rates and elevated MAPK12 levels in DLBCL patients. Based on the expression of MAPK12, DLBCL patients were split into two groups in each figure. **a-b.** PFS and OS survival analyses results for 153 patients. **c-d.** Analyses of patients survival with age ≤ 60 or > 60. **e–f.** Analyses of patients survival with ECOG ≤ 1 or > 1. **g-h.** Analyses of patients survival with normal LDH or LDH > normal. **i-j.** Analyses of patients survival with extra node < 2 or ≥ 2. **k-l.** Analyses of patient survival with Ann Arbor stage I–II or III–IV. **m–n.** Analyses of patient survival with IPI score 0–2 or 3–5. **o-p.** Analyses of patient survival with non-GCB subtype or GCB subtype

**Fig. 4:**
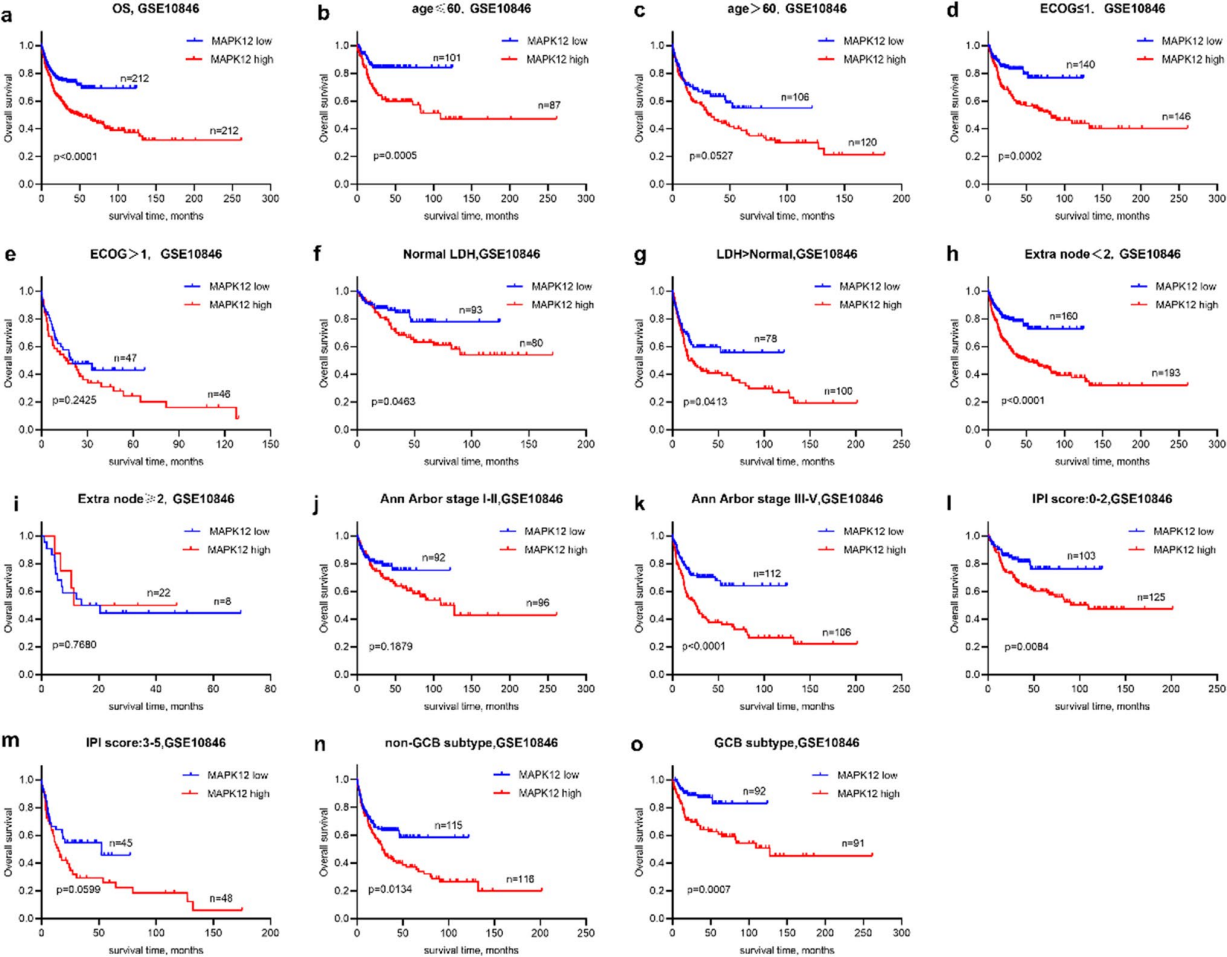
Association between MAPK12 levels and survival in GSE10846.Based on the expression of MAPK12, DLBCL patients were split into two groups in each figure. **a.** OS analysis results for 414 patients. **b-c.** Analyses of patients survival with age ≤ 60 or > 60.**d-e.** Analyses of patients survival with ECOG ≤ 1 or > 1. **f-g.** Analyses of patient survival with normal LDH or LDH > normal. **h-i**. Analyses of patient survival with extra node < 2 or ≥ 2. **j-k.** Analyses of patient survival with Ann Arbor stage I–II or III–IV. **l-m.** Analyses of patient survival with IPI score 0–2 or 3–5. **n–o.** Analyses of patient survival with non-GCB subtype or GCB subtype

Correlation analysis was conducted to investigate the correlation between MAPK12 expression and various clinical outcomes. The results revealed that patients with DLBCL who exhibited high MAPK12 expression (*n* = 66) had significantly lower survival rates compared to those with low MAPK12 expression (*n* = 87) (*p* = 0.0016) (Fig. 3a). In the high MAPK12 expression group, 45 patients (68.2%) experienced disease progression or death, whereas in the low MAPK12 expression group, 42 patients (48.3%) experienced such events. The PFS was found to be statistically significant (*p* = 0.0161) (Fig. 3b). To validate these findings, we utilized the GEO database (*n* = 414, Fig. 4a), which confirmed a correlation between survival and MAPK12 expression. According to the GEO database, patients with high MAPK12 expression had a worse survival prognosis for DLBCL.

IPI score is a recognized indicator for predicting the prognosis of DLBCL. The quantity of extranodal disease sites, age, serum LDH concentrations, AAS, and ECOG performance status are all considered. In this study, we performed subgroup survival analysis on patients to evaluate the impact of these characteristics on survival (Figs. 3c-l and 4b-k). Our research showed that patients with elevated MAPK12 expression had considerably shorter survival times. Furthermore, we found that MAPK12 expression was significantly related to OS in patients with low-risk IPI (Figs. 3m-n and 4l-m). This trend was generally consistent across the various subgroups. According to the Hans Classification, compared with non-GCB, MAPK12 expression was correlated with survival in patients with GCB (Fig. 3o-p). Our study, however, found a correlation between survival in GCB and non-GCB patients was correlated with MAPK12 expression in GSE10846 (Fig. 4n-o). Evidently, patients with higher levels of MAPK12 expression had lower survival rates.

## Prognostic factors in DLBCL: univariate and multivariate Analyses

A Cox proportional risk regression model was employed to further examine the potential prognostic value of MAPK12 expression in DLBCL patients. Univariate analysis revealed significant associations between survival and age (*p* = 0.018), ECOG score (*p* = 0.035), IPI score (*p* < 0.001), LDH (*p* = 0.007), MAPK12 expression (*p* = 0.001) and the use of Rituximab (*p* = 0.044). Multivariate analysis further confirmed that IPI score (*p* = 0.014), MAPK12 expression (*p* = 0.040), and the use of rituximab (*p* = 0.028) were independently associated with OS in DLBCL patients (Table 2). Our findings suggest that MAPK12 expression may significantly influence the prognosis of patients with DLBCL.

**Table 2:** Univariate and multivariate analysis of prognostic factors for OS in DLBCL

	Univariate analysis		Multivariate analysis	
	HR (95%CI)	P value	HR (95%CI)	P value
Gender Female vs. male	1.017 (0.625–1.657)	0.944	–	–
Age (years) > 60 vs. ≤ 60	1.822 (1.109–2.993)	0.018^*^	0.976 (0.531–1.794)	0.937
ECOG score2-4 vs. 0–1	1.767 (1.042–2.995)	0.035^*^	1.008 (0.533–1.908)	0.980
Ann Arbor stageIII–IV vs. I–II	1.635 (0.998–2.680)	0.051	–	–
IPI score3-5 vs.0–2	2.898(1.776–4.727)	< 0.001^*^	2.997 (1.254–7.164)	0.014^*^
B symptoms Present vs. absent	0.980 (0.542–1.772)	0.946	–	–
LDH(U/L) > normal vs. normal	1.980 (1.208–3.245)	0.007^*^	0.852 (0.411–1.765)	0.666
Ki–67(%) > 70 vs. ≤ 70	1.010(0.616–1.656)	0.967	–	–
No. of extranodal involvement(s) < 2 vs. ≥ 2	1.653 (0.928–2.945)	0.088	–	–
Tumor size(cm) ≥ 10 vs. < 10	1.591 (0.811–3.122)	0.177	–	–
Hans classification Non–GCB vs. GCB	1.329 (0.812–2.174)	0.257	–	–
MAPK12 expression High vs. low	2.237 (1.362–3.674)	0.001^*^	1.734 (1.025–2.935)	0.040^*^
Rituximab use vs. not	1.667 (1.014–2.740)	0.044^*^	1.776 (1.064–2.963)	0.028^*^

^*^p ≤ 0.05

## WGCNA and identification of key modules

**Fig. 5:**
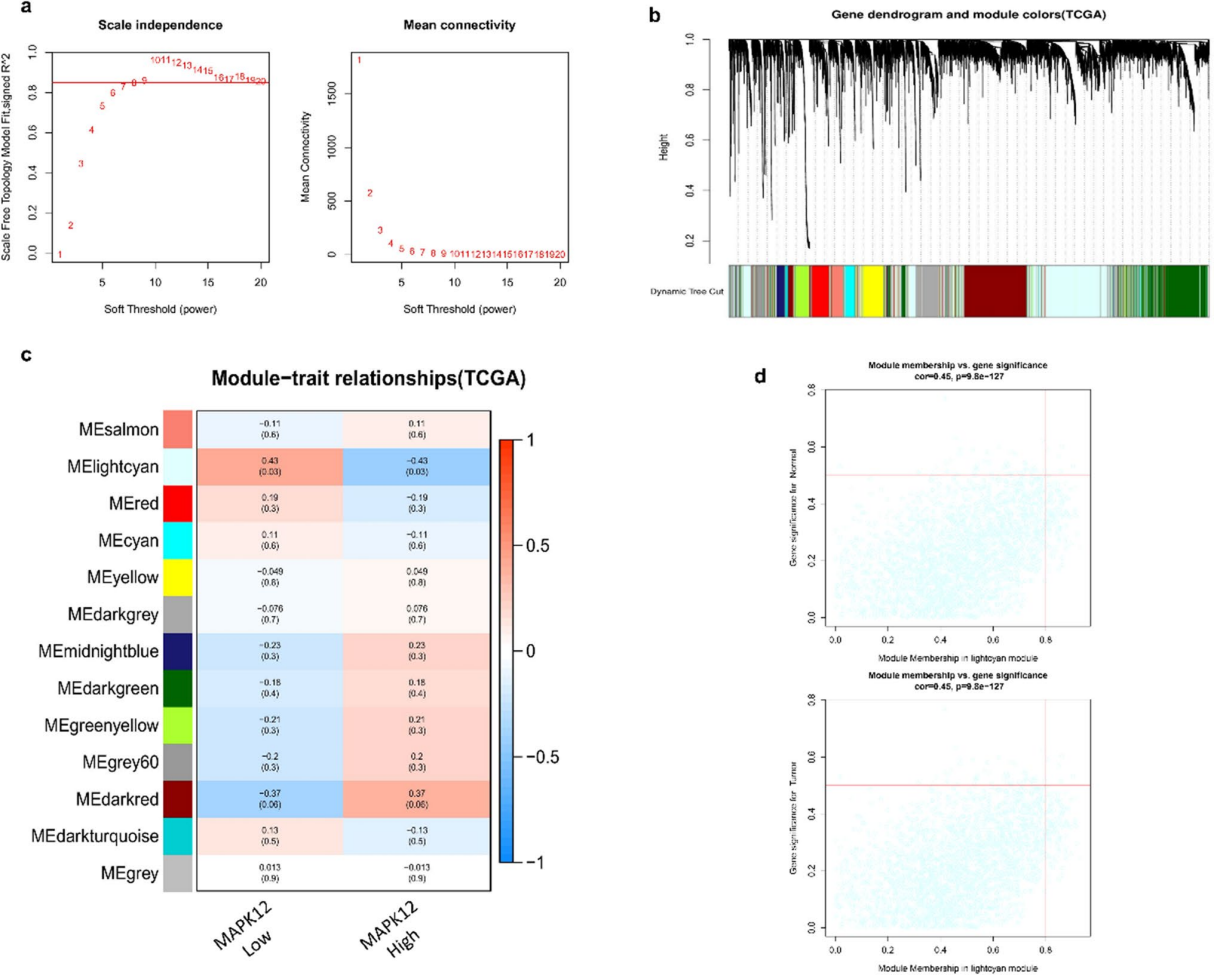
WGCNA and key module identification. **a.** The scale-free topology requirement in TCGA-DLBCL was used to determine the soft-thresholding power (β) of 9. **b.** Clustering dendrograms showed genes with similar expression patterns were clustered into co-expression modules in TCGA-DLBCL. The gray module indicates that genes were not assigned to any module. **c.** Module–trait interactions in TCGA-DLBCL demonstrate connections between each module and MAPK12 expression. **d.** Module membership (MM) and gene significance (GS) scatter plots for each gene in the TCGA-DLBCL lightcyan module. The correlation between the gene and co-expression module is shown on the horizontal axis, while the correlation between the gene and phenotype is shown on the vertical axis

A weighted correlation network analysis can be used to identify modules with strongly associated genes (WGCNA) [18]. To evaluate the functional significance of individual modules, genes with highly correlated expression patterns are grouped into the same modules, since they are expected to play similar or closely related biological roles [19]. WGCNA analysis was performed in TCGA-DLBCL. The soft threshold power (β) of 9 in TCGA-DLBCL (scale-free R2 = 0.85) was estimated to build a scale-free topology network (Fig. 5a). The dynamic hybrid cutting of the TCGA-DLBCL grouped 13 co-expression models (Fig. 5b). The lightcyan module showed the strongest correlation with MAPK12 expression (Cor = 0.43, *P* = 0.03) (Fig. 5c), which contains a total of 2537 genes. On the heat map, red indicates a positive association, whereas blue indicates a negative correlation. The scatter plots within the lightcyan module showed associations between module membership (MM) and gene significance (GS) in both normal and tumor cells (Cor = 0.45, *p* = 9.8e^−127^) (Fig. 5d).

## MAPK12-related gene enrichment analysis

**Fig. 6:**
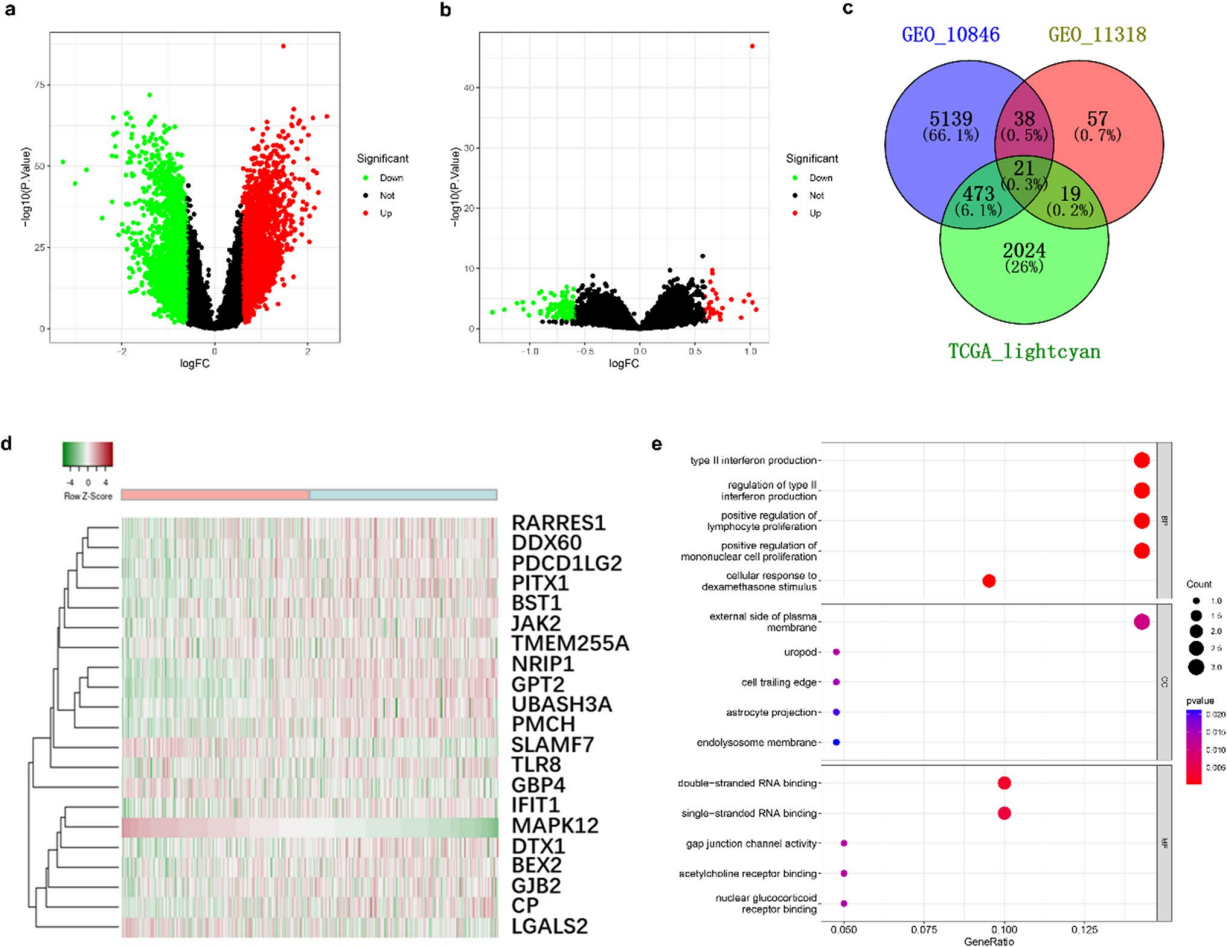
MAPK12-related gene enrichment analysis. **a.** Volcano map of DGEs in GSE10846. **b.** Volcano map of DGEs in GSE11318. **c.** The Venn diagram showed the confluence of the GSE10846 DEGs, GSE11318 DEGs, and the TCGA-DLBCL lightcyan module genes. **d.** Relationship between MAPK12 mRNA expression and the other co-expression genes is presented in the heat map. **e.** GO analysis of the enriched terms for the 21 genes' biological process (BP), cellular component (CC), and molecular function (MF)

We retrieved 420 samples from GSE10846 and 203 samples from GSE11318 in the GEO database using the R package “chip”. In order to classify the samples, we based them on their median MAPK12 expression level. In GSE10846, we identified 5,671 DEGs; and in GSE11318, we identified 135 DEGs. The DEGs were selected based on the |log_2_^Fold Change^|≥ 1.5 criteria and adjusted *P* ≤ 0.05 (Fig. 6a-b).

As depicted in Fig. 6c, 21 genes were obtained by crossing TCGA-DLBCL with GSE10846 and GSE11318. We then investigated the association of MAPK12 with these co-expressed genes. A positive correlation was observed with 17 of these genes, while a negative correlation was observed with 3 genes (Fig. 6d). Notable positive correlated genes included SLAMF7, GBP4, LGALS2, and negatively correlated genes included RARRES1, DDX60, NRIP1, and so on. Subsequently, we performed GO enrichment analysis on these genes. Type II interferon production, regulation of type II interferon production, positive regulation of lymphocyte proliferation, and mononuclear cell proliferation were the major enriched biological process (BP) terms. Moreover, the outer side of the plasma membrane was the predominantly enriched cellular component (CC) term (Fig. 6).

## Discussion

In this study, MAPK12 has been identified as a potential oncogene through both bioinformatics analysis and examination of clinical tissue samples. Furthermore, DLBCL patients have been shown to have a positive prognosis based on its presence. High expression of MAPK12 is associated with higher IPI scores, increased Ki-67 expression, and shorter PFS and OS in DLBCL patients.

According to previous studies, MAPK12 plays a crucial role in developing the digestive system and other systemic cancers. MAPK12, for example, is substantially expressed in nasopharyngeal and hepatocellular carcinoma and linked to disease prognosis [8, 20]. The mechanism involves MAPK12, acting as a CDK-like kinase, which can phosphorylate and inactivate the tumor suppressor gene RB. This leads to the promotion of cells transitioning from a quiescent state into the cell proliferation cycle and abnormal proliferation, ultimately inducing tumorigenesis. Osteosarcoma has been found to have an exact mechanism for promoting tumor growth [16]. Wang et al. found that MAPK12 links KRAS oncogene signal transduction with aerobic glycolysis, inducing the formation of pancreatic tumors [21]. Accordingly, various types of tumors might respond favorably to targeting this enzyme therapeutically. However, its carcinogenic role in DLBCL and the detailed molecular mechanism remains unclear.

In the present research, we verified that MAPK12 expression was correlated with higher IPI score, non-GCB subtype, higher LDH and higher Ki-67 using immunohistochemical staining and clinical features. These four factors were found to be correlated with the prognosis of DLBCL patients to some extent [5, 22–24]. Meanwhile, compared to non-GCB DLBCL patients, GCB DLBCL patients exhibited higher survival rates [2]. In our study, tissue samples from non-GCB DLBCL exhibited higher levels of MAPK12 expression compared to GCB DLBCL. This may suggest a connection between MAPK12 and the cancerous nature of DLBCL. We hypothesized that it might impact the prognosis of DLBCL patients.

Several prognostic factors are associated with DLBCL, including MAPK12, revealed in this retrospective study. This study is the first of its kind to propose such a possibility. Age, ECOG, IPI score, LDH, rituximab, and MAPK12 expression were associated with prognosis based on univariate analysis. Through multivariate analysis, IPI score, rituximab, and MAPK12 expression were identified as independent OS risk factors in DLBCL patients. Our findings align with previous studies that have demonstrated the influence of MAPK12 overexpression on the prognosis of patients with pancreatic [22], bladder [25], and colon cancer [26]. Additionally, using MAPK12 inhibitors has been shown to inhibit tumor formation[27, 28]. Therefore, MAPK12 could serve as a novel prognostic biomarker or diagnostic tool for DLBCL.

There is a strong correlation between high MAPK12 expression and poor prognosis in DLBCL patients. Interestingly, when grouping high-and low-risk with age, ECOG, serum LDH concentrations, the quantity of extranodal disease sites, Ann Arbor stage, IPI and non-GCB/GCB subtype, we found that MAPK12 expression was more prognostic in the low-risk group, such as low IPI scores, age ≤ 60, GCB subtype, that is, when MAPK12 was highly expressed, patients with low OS. Prior to standardized treatment, patients with low IPI scores and GCB type DLBCL, who were not double-/triple-hit lymphomas, usually had a better prognosis. However, there were still a few patients unable to achieve CR (complete response). Adding Pola (CD79B targeting medication) or glofitamab (CD3/CD20 double antibody) to 6–8 cycles of R-CHOP may improve survival rates for patients with high MAPK12 expression in the low-risk group. Our data is too small to establish the practicality of MAPK12 as a guide for therapeutic therapy selection, so larger cohort studies are needed.

Given the significance of MAPK12 expression in DLBCL, we conducted a heatmap analysis to explore the relationship between MAPK12 and its related genes. RARRES1, NRIP1, GJB2 and others showed a negative correlation with MAPK12 and might be cancer suppressor genes in DLBCL. In the literature, RARRES1 has been identified as a tumor suppressor gene in several malignancies, including renal cell carcinoma [29], follicular lymphomagenesis [30], and osteosarcoma [31]. Our research findings consistently suggest that RARRES1 may also function as a suppressor gene in DLBCL. Different tumors are inhibited by NRIP1 and GJB2 [32, 33]. Hence, we hypothesize that MAPK12 likely plays a tumor-promoting role in DLBCL.

A functional enrichment analysis of MAPK12 using GO was performed to investigate further the mechanisms by which MAPK12 promotes the occurrence and development of DLBCL. The GO analysis revealed that MAPK12 is primarily associated with regulating type II interferon production, positive regulation of lymphocyte proliferation, and positive regulation of mononuclear cell proliferation. Type II interferon, also known as IFN-γ, is crucial in immunotherapy [34]. In GCB DLBCL, when the responsiveness to IFN-γ is reduced, the interaction between lymphoma cells and the immune microenvironment is disrupted, and immune evasion occurs [35]. Rui et al. found that myeloperoxidase induced IFN-γ production by monocytes via p38γ/δ [36]. In our findings, MAPK12 was observed to positively regulate mononuclear cell proliferation. However, the molecular mechanism underlying the relationship between MAPK12 and IFN-γ in DLBCL remains unclear, and further research is needed. Another result of GO functional enrichment was positive lymphocyte proliferation regulation, consistent with Zhang's research findings [28]. Meanwhile, MAPK12 has been shown to have a promoting and carcinogenic effect on immune cells [37].

Our findings need to be confirmed by larger sample sizes in the future due to the specificity of the disease. Furthermore, we found that prognosis is associated with MAPK12 expression in DLBCL. The nature of the connection between MAPK12 and human malignancies requires clarification, given the crucial role of MAPK12 in tumor initiation and growth. This research is the first to show that MAPK12 expression indicates a poor prognosis in patients with DLBCL. It is worth noting that targeting MAPK12 could be a unique treatment approach for DLBCL.

## Data Availability

The data that support the findings of this study are available on request from the corresponding author.
